# Inducing Cross-Clade Neutralizing Antibodies against HIV-1 by Immunofocusing

**DOI:** 10.1371/journal.pone.0003937

**Published:** 2008-12-15

**Authors:** Michael Humbert, Robert A. Rasmussen, Helena Ong, Fabian M. P. Kaiser, Shiu-Lok Hu, Ruth M. Ruprecht

**Affiliations:** 1 Dana-Farber Cancer Institute, Boston, Massachusetts, United States of America; 2 Harvard Medical School, Boston, Massachusetts, United States of America; 3 University of Washington, National Primate Research Center, Seattle, Washington, United States of America; New York University School of Medicine, United States of America

## Abstract

**Background:**

Although vaccines are important in preventing viral infections by inducing neutralizing antibodies (nAbs), HIV-1 has proven to be a difficult target and escapes humoral immunity through various mechanisms. We sought to test whether HIV-1 Env mimics may serve as immunogens.

**Methodology/Principal Findings:**

Using random peptide phage display libraries, we identified the epitopes recognized by polyclonal antibodies of a rhesus monkey that had developed high-titer, broadly reactive nAbs after infection with a simian-human immunodeficiency virus (SHIV) encoding *env* of a recently transmitted HIV-1 clade C (HIV-C). Phage peptide inserts were analyzed for conformational and linear homology using computational analysis; some peptides mimicked various domains of the original HIV-C Env, such as conformational V3 loop epitopes and the conserved linear region of the gp120 C-terminus. Next, we devised a novel prime/boost strategy to test the immunogenicity of such phage-displayed peptides and primed mice only once with HIV-C gp160 DNA followed by boosting with mixtures of recombinant phages.

**Conclusions/Significance:**

This strategy, which was designed to focus the immune system on a few Env epitopes (immunofocusing), not only induced HIV-C gp160 binding antibodies and cross-clade nAbs, but also linked a conserved HIV Env region for the first time to the induction of nAbs: the C-terminus of gp120. The identification of conserved antigen mimics may lead to novel immunogens capable of inducing broadly reactive nAbs.

## Introduction

HIV-1 continues to spread and has become a pandemic with more than 34 million infected people and 14,000 new infections per day [1]. Despite intense research efforts over the last 20 years, a safe, effective vaccine against HIV-1/AIDS has not yet been found, and its development remains a top priority. To date, large-scale phase III clinical trials with candidate AIDS vaccines have been disappointing (reviewed in [2], [3]); such trials involved an attempt to generate neutralizing antibody (nAb) response-based vaccines based upon the surface subunit gp120 as well as a vaccine strategy designed to induce cytotoxic T-lymphocyte (CTL) responses with recombinant adenovirus vectors.

The viral envelope glycoproteins, non-covalently linked trimers consisting of three gp120 and three gp41 subunits, divert the immune system with variable loops which cover neutralization-sensitive Env regions [4], [5]. Env glycoproteins frequently change their amino acid sequence in response to selective pressure exerted by the immune system, thus presenting the host with ever new antigens. Furthermore, the trimeric Env structure shields important domains of the Env core, making them inaccessible to antibody-mediated neutralization [6]. Conformational Env re-orientation upon CD4 receptor binding transiently uncovers neutralization-sensitive regions for coreceptor binding until the viral envelope fuses with the host cell membrane. Additionally, heavy glycosylation on the outside of gp120 hides much of the protein core from antibody attack (reviewed in [7], [8]).

Proof-of-concept passive immunization studies in primates challenged with simian-human immunodeficiency viruses (SHIVs) yielded clear-cut evidence of the ability of several neutralizing human monoclonal antibodies (nmAbs) to provide complete protection from infection [9], [10], [11], [12], [13], [14], [15], [16], [17] (reviewed in [18]). As a consequence, the epitopes targeted by these nmAbs can be considered to be protective epitopes. The nmAbs used in passive immunization experiments also neutralized a number of primary strains of HIV-1 of different clades in vitro alone and especially in combination in different assay systems [19], [20], [21], [22], indicating their broad reactivity. The following nmAbs were involved in passive immunization studies yielding complete protection: 2G12, which binds to mannose residues on gp120 [23]; b12 or F105, antibodies against the CD4 binding site (CD4bs) [24], [25]; as well as 4E10 and 2F5, which bind to adjacent epitopes in the membrane proximal external region (MPER) of gp41 [26]. However, Haynes et al. [27] linked three out of the four human nmAbs recognizing protective epitopes to autoreactivity. These investigators demonstrated that 4E10 and to a somewhat lesser degree 2F5 cross-react with cardiolipin, a self-antigen. This observation may explain the inability to induce 4E10/2F5-like nAbs described by several groups [28], [29], since repeated boosting may eliminate autoreactive B cells. The protective epitopes may also be poorly immunogenic because they either are located in recessed regions of gp120 (the CD4bs) or are only transiently accessible (the nmAbs targeting the extracellular domain of gp41). Furthermore, the special features of b12, including an unusually long, finger-like structure, might prove to be difficult to induce with current immunization strategies [30].

To identify promising vaccine candidates, it is important to know which parts of HIV-1 Env are immunogenic and able to induce protective antibodies in the host [31]. X-ray crystallography has revealed important structural Env features and sites of interaction with cellular receptors; it is becoming evident that conserved Env parts are hidden from the immune system [8], [32]. Thus far, however, this important information has not yet been translated into a potent vaccine.

Valuable sources to study natural immune responses against HIV-1 Env are sera with high-titer, cross-clade nAbs. The analysis of such antibody responses might give important information regarding structures on HIV-1 Envs that are conserved across clades. We have identified a cohort of rhesus macaques infected with SHIV-1157ip, a chimera that encodes *env* of a recently transmitted HIV-C strain, or the related SHIV-1157ipd3N4 [33] that developed high-titer nAb responses against homologous SHIV-C as well as heterologous primary strains of HIV-1 of different clades. We have employed phage display to identify the HIV-C Env structures recognized by such broadly reactive sera.

Phage display [34] is a widely used technique to analyze humoral immune responses [35], [36], [37], [38], [39], [40], [41], to map antibody epitopes [42], [43], [44], [45] or to study protein interaction sites in general [46]. Until recently, the identification of conformational epitopes was limited due to difficulties in projecting the linear mimotope sequence onto a protein structure. In the last years, attempts at closing this gap involved the development of software [47], [48], [49], [50], [51], [52] that allows three-dimensional (3D) analysis. These programs project the linear peptide sequence onto the 3D surface structure of target proteins by using published protein structure files. 3DEX [52] maps conformational mimotopes in 3D protein structures by using an algorithm that takes into account the physicochemical neighborhood of individual amino acids. A discontinuous epitope is localized within the 3D protein structure by searching for a 3D fit with partial amino acid strings of a given mimotope in a pre-set distance on the protein surface. This algorithm is repeated for each string of amino acids until the full peptide sequence is analyzed.

We dissected the humoral immune response of rhesus monkeys with broadly reactive nAb responses using phage display; recombinant phage peptide sequences were evaluated for conformational and linear homologies to gp160 using computational analysis [52]. Phage peptides encoding mimotopes were used to isolate the cognate antibodies from polyclonal rhesus monkey serum; these affinity-purified antibodies were then tested for differential ability to recognize native versus denatured HIV-1 Env. Promising mimotopes were used in a novel DNA prime/phage boost immunization strategy aimed at focusing the antibody response on the regions represented by mimotopes. This vaccination yielded cross-clade nabs responses in immunized mice.

## Results

### Selection of HIV-C Env-Specific Mimotopes

We used polyclonal IgG from a rhesus monkey infected with SHIV-1157ip, an R5 SHIV strain encoding *env* of a recently transmitted Zambian HIV-C. This monkey (animal RKl-8) as well as others of our cohort had developed high-titer, broadly reactive nAbs that neutralized primary strains of HIV-1 and SHIV of clades B and C (Table 1). High nAb titers were detected against the early SHIV-C (SHIV-1157ip), the late isolate SHIV-1157ipd3N4 [33], as well as against a heterologous R5 SHIV-C generated in our group (SHIV-2873Nip). All sera were tested against a panel of heterologous HIV clade C and B strains (Table 1) and showed cross-clade nAbs against various strains, including HIV_pIndieC_, HIV_SF162.LS_ and HIV_NL4-3_, especially serum of monkey RKl-8. Tissue culture supernatants of B-lymphocytic cell lines generated from monkey RKl-8 showed neutralizing activity against various SHIV and primary HIV strains, including clades A, B and C (data not shown). Serum IgG was immobilized on paramagnetic beads to isolate specific mimotopes by screening three different phage-displayed random peptide libraries (7mer, cyclic 7mer, 12mer). For each screening, 94 single clones were tested in phage ELISA for their specificity using SHIV-positive and SHIV-negative serum in parallel. Positive clones were amplified and sequenced. Peptide insert sequences were grouped according to their motifs and analyzed for linear homology to parental gp160_SHIV-1157ip_. Using monkey RKl-8 serum [33], we isolated 78 different clones; for gp120, we identified mimotopes representing the V2 loop (9 clones), the V3 loop (21 clones) and the C-terminal domain (8 clones). Thirty-four of the 78 clones resembled regions on gp41: The majority of the clones represented a subdomain of the immunodominant region (IDR), which is referred to as the immunodominant loop [31] and contains the KLIC motif (20 clones); seven clones shared homology with IDR outside the immunodominant loop as well as several amino acid residues of the N-terminal heptad repeat. Seven other clones showed homology to the MPER. Finally, six phage inserts exhibited no apparent linear similarity to gp160_SHIV-1157ip_.

**Table 1:** IC_50_ * for selected rhesus monkeys with high-titer nAb activity.

Animal #	Homologous Clade C		Heterologous Clade C					Heterologous Clade B		
	SHIV-1157ip (early)^1^	SHIV-1157ipd3N4 (late)^2^	HIV_pIndieC_ 1	ZM135M^1^	ZM233M.PB6^1^	ZM109F^1^	SHIV-2873Nip^2^	SHIV_SF162P3_ 1	HIV_SF162.LS_ 1	HIV_NL4-3_ 1
RKl-8^3^	2,048	>640	2,048	24	160	26	>640	96	3,741	512
RAo-8^3^	2,048	>640	128	<20	42	<20	>640	<40	220	32
RJa-9^3^	1,800	>10,240	128	<20	59	22	2,048	68	35,770	128
RMf-9^3^	2,048	>640	128	<20	<20	35	>640	78	18,303	128
RTs-7	2,048	>640	128	n.d.	n.d.	n.d.	>640	n.d.	n.d.	32
RHy-9	600	>10,240	90	<20	<20	<20	>640	n.d.	173	n.d.

* IC_50_, 50% inhibitory concentration given as reciprocal serum dilution for 50% neutralization.

1 determined in TZM-bl assays.

2 determined in human PBMC-based assays.

3 phage display selection performed; n.d. not determined.

ZM135M, ZM233M.PB6, and ZM109F are primary HIV clade C isolates from Zambia. No neutralization was seen against five HIV clade C isolates from South Africa and four others from Zambia (not shown). SHIV-2873Nip is a Tier 1 (i.e. neutralization-sensitive) R5 virus that encodes *env* of a recently transmitted, R5 HIV clade C isolated from a Zambian infant [71]. SHIV_SF162P3_ is a Tier 2 virus (i.e. more difficult to neutralize and representative of most primary HIV isolates), HIV_SF162.LS_ is a Tier 1 virus.

**Figure 1:**
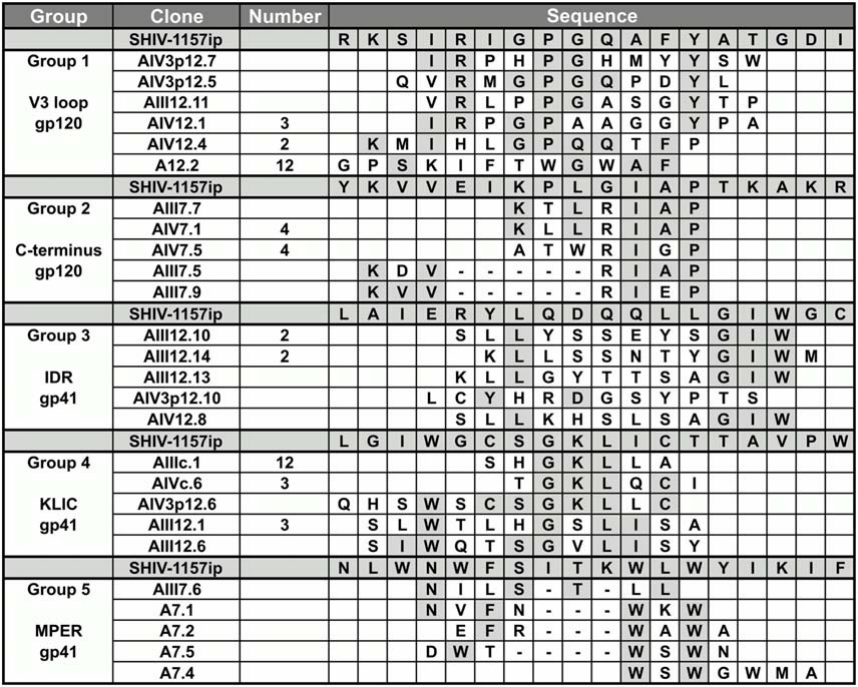
Alignment of mimotopes selected with serum from monkey RKl-8 with the sequence of homologous gp160_SHIV-1157ip_. Phage peptide sequences (clones) were grouped according to their motifs (V3, gp120 C-terminus, gp41 immunodominant region (IDR), KLIC, and membrane proximal external region (MPER)) and aligned to gp160_SHIV-1157ip_ (grey rows). Linear homologies are shaded grey. Numbers in parentheses indicate how many times a given mimotope was selected independently. In a working definition, mimotopes were considered linear if they exhibited more than 50% linear amino acid identity.

**Figure 2:**
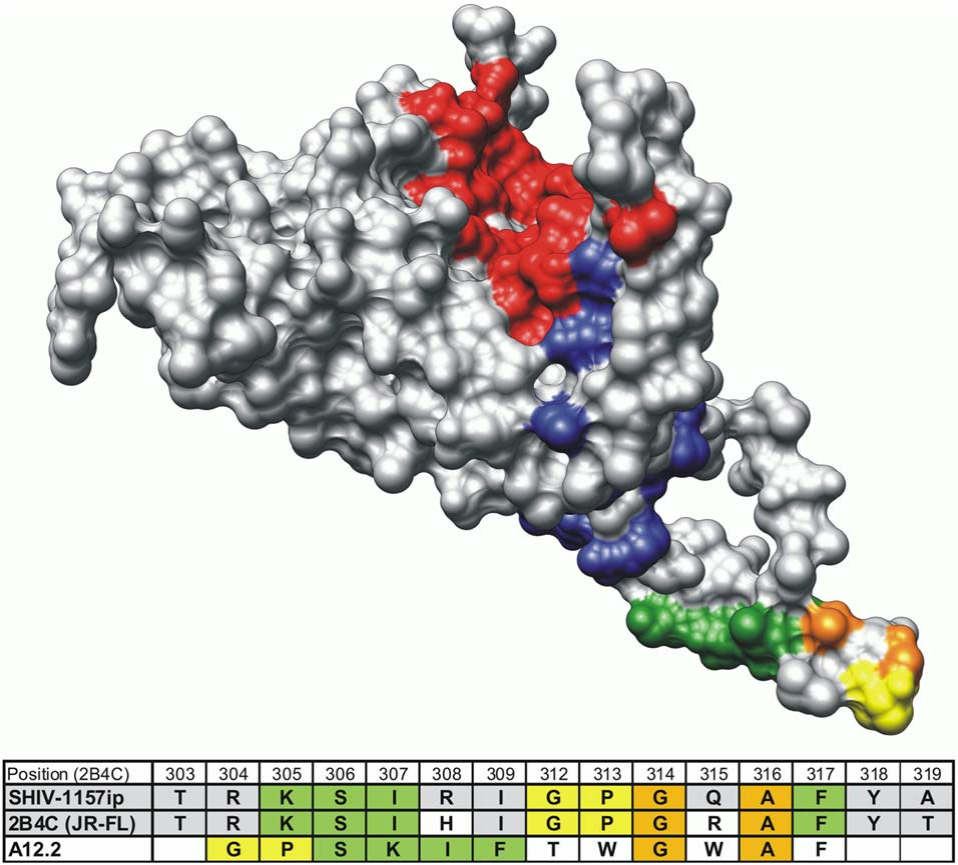
Location of mimotope A12.2 on gp120. 3DEX analysis [52] was used to find structural homology between A12.2 (yellow, orange and green) and the surface of gp120 (PDB ID: 2B4C) with the CD4 binding site shown in red and CCR5 coreceptor contact sites in blue. The inset table shows partial V3 sequences for SHIV-1157ip, the protein structure used for the 3DEX analysis (2B4C), and A12.2. Amino acid residues resembling the parental sequence but too few to form a linear epitope are shown in yellow and orange. Residues identified by 3DEX as showing 3D homology are shown in green. All amino acids are in close proximity on the molecule's surface and form a potential conformational mimotope of gp120 (Figure prepared with Chimera [70]).

Since several mimotopes displayed only minimal linear homology to gp160_SHIV-1157ip_, we postulated that these exhibit predominantly conformational homology. Using the above mentioned software 3DEX [52] and a published gp120 structure [53], we sought to identify conformational mimotopes. This approach allowed us to identify an interesting V3 mimotope which combines linear with structural homology (Figure 1, clone A12.2; Figure 2). We compared the primary envelope sequence of the structure file (PDB-ID: 2B4C; HIV-1 subtype B strain JR-FL) and SHIV-1157ip. The amino acids in that stretch of V3 are almost identical in sequence (inset Figure 2). 3DEX found two motifs: one that comprises four amino acids at the crown of the V3 loop (Figure 2, yellow/orange); and a second one that consists of another four amino acids located near the crown (Figure 2, green). Even though these eight amino acids are discontinuous, they are found in neighboring locations at the Env surface.

### Identification of a Conformational Mimotope of HIV-C Env

**Figure 3:**
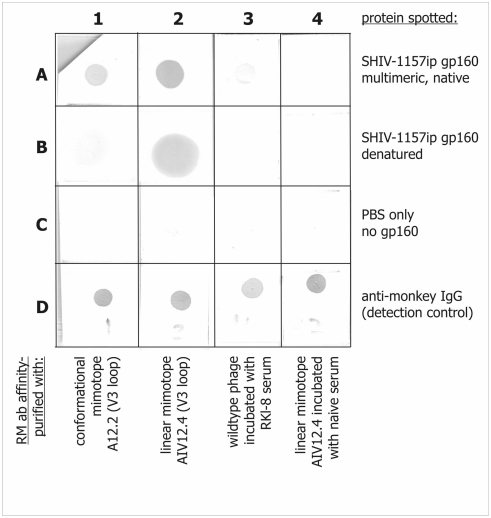
The conformational dependence of mimotope A12.2 by dot spot analysis with phage affinity-purified serum antibodies. The following were spotted onto the filter: row A, native gp160_SHIV-1157ip_; row B, denatured gp160_SHIV-1157ip_; row C, no protein spotted; row D, anti-rhesus monkey IgG. In columns 1–4, rhesus monkey antibodies affinity-purified with the following reagents were applied: (1) recombinant phage A12.2 encoding mimotope identified as conformational by 3DEX; (2) phage AIV12.4 encoding linear mimotope; (3) WT phage; and (4) AIV12.4 incubated with naïve rhesus monkey serum.

To confirm that antibody binding to mimotope A12.2 involves interaction with a conformational epitope and thus depends on the structural integrity of the target HIV-C gp160, we compared antibody binding to the region on HIV-1 Env represented by the mimotope under native and reduced conditions. First, we used affinity purification with immobilized recombinant phage to isolate the cognate antibodies from the polyclonal rhesus monkey serum. The phage-affinity-purified antibodies were then subjected to a dot spot analysis with homologous, trimeric gp160_SHIV-1157ip_ immobilized under native and denaturing conditions (Figure 3). Binding of antibodies specific for clone A12.2 was abolished when trimeric gp160 was denatured (Figure 3, field A1 and B1), although specific binding was demonstrated to native Env. As control, we used antibodies specific for a phage clone with greater linear homology to the V3 loop (AIV12.4); as expected, these antibodies recognized the native as well as the denatured forms of gp160 (Figure 3, field A2 and B2). The control without spotted gp160 (Figure 3, row C) showed no nonspecific antibody binding on any strips. An additional positive control involved spotting anti-monkey IgG (Figure 3, row D) and confirmed that equal amounts of phage-affinity-purified antibodies had been applied to all strips.

### Reactivity Profile of Mimotopes in the Context of Fusion Proteins

To analyze if the HIV-1 Env mimotope motif groups represent common antibody epitopes found in sera with broadly reactive nAbs, we cloned selected mimotopes of all groups (except MPER) into fusion proteins and tested the latter by ELISA and Western blot analysis using a panel of rhesus monkey sera with broadly neutralizing activity against different HIV-1 clades (Table 1). All monkeys show broadly reactive nAbs against the homologous SHIV-1157ip as well as against a heterologous HIV-1 clade C (pIndieC) and clade B (HIV_SF162.LS_). In addition to screening with serum from monkey RKl-8 described above, sera from monkeys RAo-8, RJa-9 and RMf-9 were used for individual phage display selections. The analysis revealed similar motif patterns to RKl-8, including mimotopes representing immunodominant regions such as V3 and KLIC, but we also selected new motifs representing stretches of the Kennedy peptide [54] in gp41 and as yet unidentified regions (data not shown).

**Figure 4:**
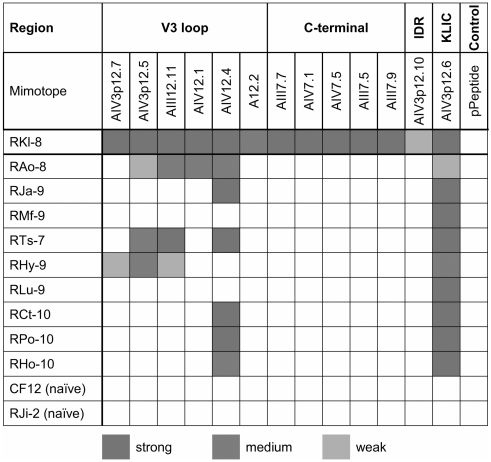
Cross-reactivity of mimotope fusion proteins with rhesus monkey sera showing broadly reactive nAbs. Mimotopes were tested in the context of fusion proteins against 11 rhesus monkey sera for binding reactivity by ELISA (see Figure S1). The binding pattern is summarized using a color code for weak (orange), medium (blue) and strong (red) binding.

**Figure 5:**
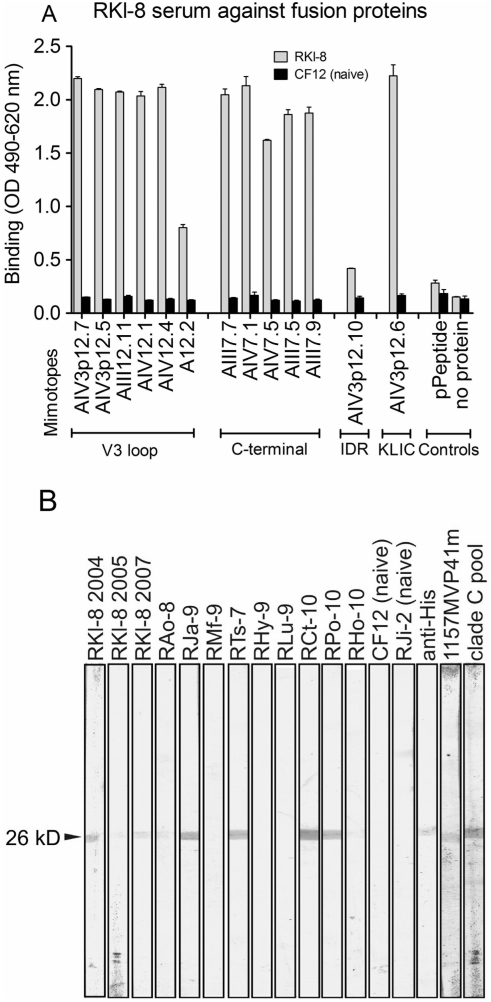
Reactivity profile of mimotope fusion proteins by ELISA and Western blot. (A) Mimotope fusion proteins were tested by ELISA with sera from monkeys RKl-8 and CF12. (B) Cross-reactivity of mimotope fusion protein AIV12.4 (26 kD; arrowhead) with a panel of rhesus monkey sera by Western blot. Two naïve sera (CF12, RJi-2) and a fusion protein without mimotope insert (pPeptide) were used as controls. Positive identification of the fusion proteins was via anti-His tag antibody. Two human sera were used for additional testing: serum from the infected source person harboring HIV-C, strain 1157iMVP41m, and pooled sera from HIV-C-infected individuals.

To assess if the mimotopes in the context of a fusion protein have conserved their structure, we performed ELISAs with sera from RKl-8. All mimotope fusion proteins are recognized by serum antibodies of RKl-8 (Figure 4, Figure 5A). Of note, there is also binding to the conformational mimotope A12.2. The binding pattern for RKl-8 was confirmed with sera taken at different time points between 2003 and 2007 (data not shown). As summarized in Figure 4, we extended the fusion protein ELISA and tested for reactivity using individual serum samples from 11 rhesus monkeys that included nine other monkeys with broadly reactive nAbs and two naïve animals (Figure S1). As expected, we observed comprehensive cross-reactivity of the KLIC mimotope with most of the sera from our monkey cohort with high-level nAbs. Notably, several rhesus monkey sera recognized selected V3 mimics, especially the two mimotopes AIV3p12.5 and AIV12.4 (Figure 4, Figure S1). Motifs isolated with serum from monkey RKl-8 representing the conserved C-terminus of gp120 and the IDR did not react with sera from our rhesus monkey panel, although reactivity was seen with autologous serum. Two naïve control sera did not detect the fusion proteins and a control fusion protein (pPeptide; empty backbone of fusion protein without mimotope insert) was not detected by any of the rhesus monkey sera.

To confirm the ELISA results, we tested all rhesus monkey sera for specific binding to the fusion proteins by Western blots. As an example, we show the result for fusion protein AIV12.4, which was broadly recognized by ELISA (Figure S1, Figure 5B). RKl-8 sera from various time points detected this mimotope as well as several other rhesus monkey sera from our panel. The naïve control sera CF12 and RJi-2 did not bind to the mimotope fusion protein, whereas the positive anti-His antibody control detected the fusion protein migrating at a MW of 26 kD. We also tested the parental serum from the HIV1157i-infected human (1157MVP41m) and a serum pool of HIV-C-infected individuals; both recognized the mimotope (Figure 5B). Interestingly, the reactivity of RKl-8 serum samples taken from different years with AIV12.4 varied as this animal progressed to AIDS [33], [55]. The early time point in 2004 showed a high reactivity with this linear mimotope in Western blot and ELISA (Figure 5B, Figure S2). However, the samples taken at later time points (2005 and 2007) showed a significant decrease in antibody binding to this mimotope. As this animal eventually progressed to AIDS, the antibody response to this protein epitope on Env may have been compromised by the reduced numbers of T-helper cells.

The ELISA and Western blot results show that the mimotopes expressed in the context of a fusion protein conserved their structure and were recognized by the monkey serum from which they were selected as well as by other sera from our cohort.

### Immunization Studies in Mice

To assess if the mimotopes representing different regions of HIV Env are immunogenic, we performed a vaccine study in mice. We used one DNA inoculation to prime the immune system with the entire HIV-1 Env, followed by phage boosting to focus the antibody response to a certain area of gp160.

**Figure 6:**
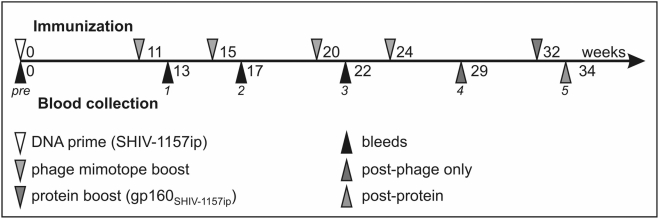
Schedule for immunizations and blood collections in mouse study. After collecting the initial pre-bleeds, all mice were primed with SHIV-1157ip *env* DNA (white triangle; Methods) followed by four phage boosts (grey triangles) every 4–5 weeks. Blood samples were collected 2–5 weeks after each boost (black/red/orange triangles). For a pilot test, 11 mice were selected to receive a final boost with trimeric gp160_SHIV-1157ip_ (green triangle).

We grouped recombinant phages according to their peptide motifs and combined selected phages into five mixtures to immunize mice (Figure 1). First, all mice received one priming immunization with a DNA vector encoding gp160_SHIV-1157ip_; this single DNA inoculation was previously shown to be insufficient for induction of binding antibodies or nAbs [56]. After one DNA prime, all mice were boosted four times with phage particles (intervals of 4–5 weeks) (Figure 6) and their immune sera were assessed for binding abs and nAbs.

**Figure 7:**
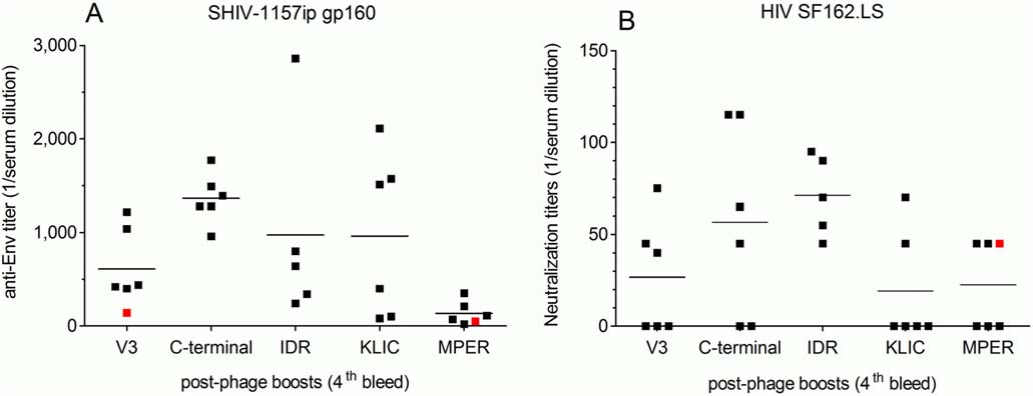
Analysis of post-phage boost (4^th^ bleed) mouse immune sera. (A) Anti-Env titers. Reciprocal serum dilution of each mouse for is shown. (B) Neutralization. Bleeds were tested for 50% neutralization (IC_50_) against heterologous HIV-1_SF162.LS_. IC_50_ values of each group are shown. Groups represent mimotopes from the V3 loop (V3) and C-terminal domain of gp120 (C-terminal); immunodominant (IDR), KLIC, and membrane proximal external region (MPER) of gp41. Red symbols indicate the use of the 3^rd^ bleed due to limited serum availability.

First, we measured antibody binding titers for each group against homologous gp160_SHIV-1157ip_ by ELISA (Figure 7A) after the four phage boosts. Mean titers ranged from 1∶125 to 1∶1362, and almost all mice developed anti-Env titers. The lowest titer was observed in the MPER group, the highest in the C-terminal domain. However, antibody binding to HIV-1 gp160 does not correlate with virus neutralization. Therefore, post-phage boost sera (4^th^ bleed) were tested for neutralization of a heterologous HIV-1 clade B strain, HIV_SF162.LS_; 59% of the animals had measurable, cross-clade anti-HIV-1 nAbs (Figure 7B). The mean IC_50_ in all groups ranged from 1∶19 to 1∶70. Of note, four out of the six mice immunized with mimotopes representing the C-terminal domain had nAbs, including two animals with IC_50_ values>1∶100. To our knowledge, this is the first report of the C-terminus of gp120 being linked to the induction of nAb responses.

To examine whether DNA prime/recombinant phage boosts had induced maximal nAb responses or if a boost with native, multimeric gp160 would be beneficial, we boosted 11 selected mice (6 from the V3, 1 from the C-terminal, 2 from each, the IDR and KLIC group) with gp160_SHIV-1157ip_ and reassessed the immune sera in comparison to those obtained after phage-boosts only. Mean Env-ELISA titers were 1∶1,220 after four phage boosts compared to 1∶2,748 after the additional gp160 boost, an increase that did not reach statistical significance (p = 0.1574; Figure S3A). When tested for nAb responses against homologous SHIV-1157ip or heterologous HIV-1_SF162.LS_, the additional Env boost did not significantly raise nAb titers against either virus (p = 0.8544 and p = 0.3935, respectively; Figure S3B), implying that maximal responses had been induced by DNA prime/phage boosting. Thus, the higher ELISA antibody titer after protein boosting did not translate into better nAb titers.

### Analysis of Vaccination-Induced Antibodies

Next, we sought to test whether antibodies induced by the different immunogens reacted differently with native versus denatured HIV-1 gp160. It is possible that the mixture of similar but not identical mimotopes was able to broaden the immune response and to induce antibodies against conformational epitopes rather than linear ones. Our V3 mimotopes show incomplete linear homology to gp160 and may contain structure-specific homologies. In contrast, the gp120 C-terminal mimotopes are linear.

**Figure 8:**
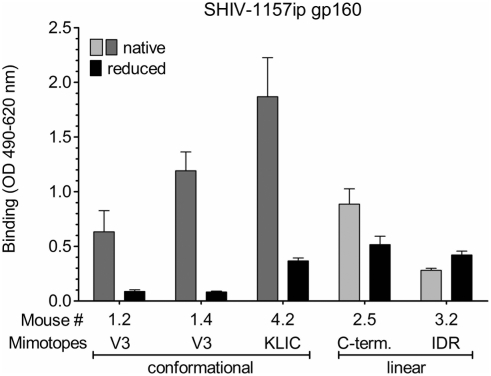
Reducing Env ELISA. Mouse immune sera were tested for binding to native (blue/orange bars) and reduced Env (black bars). Three mice were immunized with potential conformational mimotopes (#1.2, 1.4 and 4.2; blue), two mice with linear mimotopes (#2.5 and 3.2; orange).

We tested the post-protein boost sera (5^th^ bleed) for reactivity against native and reduced HIV-1 gp160 by ELISA (Figure 8). Two sera from mice boosted with V3-loop mimotopes showed decreased binding upon denaturation of Env (7–14-fold). In contrast, the signal obtained with serum from the mouse boosted with mimotopes representing the HIV-1 gp120 C-terminus decreased only 1.7-fold. These data imply that the V3-loop mimotopes induced conformational antibody responses, whereas the gp120 C-terminal mimotopes induced predominantly linear antibody responses.

Mimotope-induced antibodies against the gp41 IDR (Figure 8; mouse #3.2) showed slightly increased binding upon denaturation of gp160_SHIV-1157ip_; whereas serum antibodies from mouse #4.2 immunized with the potentially conformational KLIC motifs exhibited decreased binding after target denaturation (Figure 8), implying that they are specific for conformational epitopes.

### Anti-Mimotope Antibodies

To test if the last protein boost augmented the immune response against the actual mimotopes, we cloned all V3 and C-terminal mimotopes into expression vectors and generated fusion proteins for ELISA analysis. Due to limited availability of mouse sera, we selected one mouse from each group and tested the 4^th^ and 5^th^ bleeds (#1.4 and #2.5) (Figure S3C, S3D). Interestingly, the protein boost did not increase binding antibody titers against the six mimotopes representing the V3 loop (Figure S3C). In contrast, the HIV gp160 boost significantly increased the immune response against the linear mimotopes from the C-terminal gp120 domain by 6 to 9-fold (Figure S3D). This suggests that the conformational V3-loop mimotopes induced structure-specific antibody responses that were stimulated maximally by the one-time DNA priming followed by recombinant phage boosting. As mentioned above, the additional Env boost did not increase nAb titers.

These results support data presented in Figure 8 that mainly linear epitopes were boosted by gp160, resulting in higher binding antibody titers. However, gp160 boost-induced antibodies did not significantly improve the neutralization capacity as shown in Figure S3B. Potentially conformational anti-mimotope responses (as represented by the V3 and KLIC groups) were not boosted by the last protein boost (Figure S3C; data for KLIC not shown). It seems our DNA prime/phage boost strategy did achieve the maximum in nAb titers, although the binding titers showed modest but insignificant increases after the last protein boost.

## Discussion

Here we have shown: 1) the isolation of mimotopes specific for HIV-C Env, selected with serum from a clade C SHIV-infected rhesus monkey with broadly reactive nAbs against different clades of HIV; 2) using the 3D-structure of the clade B HIV_JR-FL_ in conjunction with 3DEX software analysis, we found Env mimics that reflect structurally conserved elements and/or conserved amino acid stretches, including motifs representing V3 and the C-terminal domain of gp120, as well as the IDR, the KLIC motif, and MPER of gp41; 3) 3DEX identified a conformational V3 mimotope with only minimal linear sequence homology. The conformational characteristics were confirmed experimentally by differential recognition of native and denatured Env using phage-affinity purified antibodies specific for this V3 loop mimotope; 4) DNA priming with a HIV-C gp160-encoding vector followed by repeated boosting with mimotope-encoding phages induced antibodies in mice that specifically bound to HIV gp160 and neutralized viruses with clade B and C envelopes.

The key for a successful mimotope lies in the possibility of its identification. Earlier attempts at identifying Env mimotopes only had one tool available: sequencing of the peptide insert. An important advance that finally allows identification based upon 3D structure was the development of the 3DEX and similar software [47], [49], [50], [51], [52]. We have given proof-of-concept for this new approach with mimotope A12.2 and have brought the finding full-circle by demonstrating differential recognition of native versus denatured HIV-C gp160 by phage affinity-purified rhesus monkey abs. Our data demonstrate that the use of random peptide phage display to select disease-specific mimotopes from a large library in combination with computational analysis may lead to the identification of novel immunogens.

For one V3 mimotope (A12.2), we could show that gp160 conformation is important for binding of its cognate phage affinity-purified antibody. The V3 loop is thought to adopt a highly specific and conserved structure to mediate coreceptor interaction [57], [58]. Thus, even if variable in its sequence, the specificity of the loop in its selective interaction may be a target for antibody-mediated neutralization [59], [60]. Our identified mimotopes may represent conformational mimics of this loop and were able to induce nAbs in mice.

Since biopanning using sera from other monkeys that also exhibited broad neutralizing activity resulted in the isolation of phage peptides similar to those selected with serum from animal RKl-8, we tested their cross-recognition pattern by ELISA using mimotope fusion proteins. Surprisingly, we did not see cross-recognition of the conserved C-terminal domain mimotopes, but several mimotopes of the V3 loop showed specific binding to other monkey sera. Cross-recognition of epitopes or mimotopes by various sera from HIV-infected individuals showing high nAb-titers is frequently equated with its potential ability to induce effective abs. Phage display with HIV-positive sera primarily results in the identification of immunodominant regions, such as KLIC, because of the abundance of antibodies targeting this region [41]. However, the frequency with which an epitope is recognized is not linked to neutralization and mimotopes with no or limited cross-reactivity may be of interest to nAb generation. The rhesus monkeys in our cohort may have developed an unusually effective immune response against Env in the context of natural infection, perhaps due to the fact that our SHIV-C, SHIV-1157ip, encodes the *env* gene of a recently transmitted virus. Interestingly, newly infected partners from a cohort of HIV discordant couples not only harbored HIV-C strains that were more easily neutralized than contemporaneous virus from the infected source person, but the recently transmitted HIV-C isolated also had shorter V-loops [61], [62]. It is possible that Env molecules of recently transmitted HIV-C have a more open configuration that favors the induction of broadly reactive nAbs. The broad neutralization reactivity of our rhesus monkey sera could be useful in identifying non-immunodominant but structurally conserved domains. Even though in the context of a natural infection this may be a rare response and not occur in each host, it could potentially be induced with epitope mimics which focus the immune response only to the part linked to nAbs.

Previous studies have shown that whole phage particles can be used to induce peptide-specific antibodies and protective immune responses in animals [37], [63], [64]. Our immunization with mimotope mixtures differs in a significant way from these earlier studies, which showed limited efficacy. Instead of using a series of multiple immunizations with recombinant phages expressing mimotopes as used by this group, we employed a novel DNA prime/recombinant phage boost strategy. We chose to use only a single priming with a DNA vector encoding the entire gp160, which will not generate binding or nAb responses [56]; we reasoned that a single DNA priming would imprint the immune system with the correct image of native gp160 structures, without diverting ab responses to immunodominant but ultimately unimportant Env regions. Instead, we hypothesized that our boosting with mimotopes would allow us to manipulate humoral immune responses by focusing them on important structural domains. This promising immunofocusing approach was shown to be effective in eliciting anti-Env abs. The immunization regimen consists of immunogens which focus the immune response on one or a few neutralizing epitopes. This potentially results in a larger proportion and higher titer of nAbs. Even though the use of selected epitopes or mimotopes as vaccines to focus the immune response on neutralizing domains is still in its beginning, several examples in the last years show the successful induction of nAbs against V2 and/or V3 [65], [66], [67]. We chose to perform the boosts with five different mimotope groups, each representing a well-defined Env region (the V3 loop, the C-terminus of gp120, gp41 IDR, and separately, the KLIC motif, and MPER). We hoped that our reductionist's approach would give us a chance to identify novel HIV Env regions capable of inducing nAbs in the absence of the otherwise overpowering influence of immunodominant regions. Indeed, this strategy was successful: for the first time, the C-terminal domain of gp120, previously identified as immunogenic but never linked to nAb responses [68], [69], was shown to induce nAbs. Interestingly, our DNA prime/phage mimotope boosts strategy appeared to have induced the maximal nAb responsiveness to a given domain, since additional boosting with the native, multimeric gp160 failed to significantly raise the nAb titers.

Our results show that the combination of phage display and 3DEX analysis is a powerful tool to analyze the humoral immune response by identifying novel antigenic mimics that represent functionally conserved Env domains, such as V3 or the C-terminal domain of gp120. Using an innovative DNA prime/phage boost regimen, we induced cross-clade nAbs against HIV clades B and C and for the first time, linked the C-terminal domain of gp120 to antibody-mediated neutralization in mice.

## Materials and Methods

### Animals

Indian-origin rhesus monkeys (*Macaca mulatta*) were housed at the Yerkes National Primate Research Center (YNPRC), Atlanta, Georgia, USA, a facility fully accredited by the Association for Assessment and Accreditation of Laboratory Animal Care International (AAALAC). All procedures were approved by the Animal Care and Use Committees of Emory University and the Dana-Farber Cancer Institute.

Female BALB/c mice (*Mus musculus*) were purchased from Taconic Farms (Germantown, NY) and enrolled in the study at 6–8 weeks of age and housed at the Harvard School of Public Health, Boston, Massachusetts, USA, a facility fully accredited by the Association for Assessment and Accreditation of Laboratory Animal Care International (AAALAC). All procedures were performed in accordance with the guidelines of the Animal Care and Use Committees of Harvard Medical School and the Dana-Farber Cancer Institute.

### Monkey serum

Serum for this study was collected from rhesus monkey RKl-8, an animal described earlier [33]. Briefly, this monkey was infected with a pathogenic R5 SHIV, termed SHIV-1157ip, which encodes the *env* gene of HIV-C, the most prevalent HIV-1 subtype worldwide. Approximately 10% of the rhesus monkeys infected with SHIV-1157ip or the related SHIV-1157ipd3N4 developed high-titer, broadly reactive nAbs not only against homologous SHIV-1157ip and the corresponding primary HIV-C, but also against heterologous strains of HIV-1 clades B and C (Table 1). These monkeys had been used in various experiments (rapid animal-to-animal passage to adapt the parental construct to rhesus monkeys, viral titrations, and vaccine challenge studies)[55].

### Phage display biopanning

Paramagnetic beads (Dynabeads M-280 tosyl activated; Invitrogen, Carlsbad CA, USA) were coated with a rabbit anti-monkey IgG (Sigma-Aldrich, St. Louis MO, USA) according to the manufacturer's instructions. Coated beads were pre-incubated while rotating for 2 h at room temperature with rhesus monkey serum (1∶250 in phosphate buffered saline/0.25% gelatin, PBSG; Gibco-Invitrogen, Grand Island NY, USA; Fisher Scientific, Fair Lawn NJ, USA). Beads were washed 5× with PBSG/0.5% (w/v) Tween-20 (PBSGT; Sigma-Aldrich) and then incubated while rotating overnight at 4°C with 10 µl of the original phage-displayed peptide library (New England Biolabs, Ipswich MA, USA). Biopannings of all three libraries (7mer, cyclic 7mer and 12mer) were performed in parallel using separate tubes. The next day, beads were washed 10× with 1 ml PBSGT and bound phages were eluted by pH shift with 0.2 M glycine-HCl pH 2.2 supplemented with 1 mg/ml BSA (Sigma-Aldrich). After neutralization with 1 M Tris-HCl pH 9.1 (Sigma-Aldrich), eluted phages were subjected to negative selection as described above using pooled sera from non-infected control monkeys. Phages remaining from the negative selections were amplified in *Escherichia coli* (ER2738, New England Biolabs), precipitated overnight at 4°C (20% PEG-8000/2.5 M NaCl; Fisher Scientific) and used for a second and third round of selection. After the third positive selection, the phages were titered, and single clones were picked and tested by phage ELISA for specific binding. Positives clones were amplified and sequenced to deduce their peptide insert.

### Phage ELISA

Plates (Greiner-Bio-One GmbH, Frickenhausen, Germany) were coated overnight at 4°C with 100 µl/well serum (1∶5,000 in carbonate-bicarbonate buffer; Sigma-Aldrich). The next day, plates were blocked (1 h, room temperature) with 200 µl/well 3% casein (Sigma-Aldrich) in PBS/0.5% Tween-20 (PBSCT) and washed 3× with 300 µl dH_2_O in an automated plate washer (BioTek Instruments, Inc., Winooski VT, USA). Then, 70 µl of control or positively selected phages that had been amplified overnight were added to 30 µl PBSCT and incubated overnight at 4°C. Plates were washed 3× and incubated for 1 h at RT with 100 µl/well of an anti-phage horse-radish peroxidase (HRP)-conjugated antibody (1∶2,000 in PBSCT; GE Healthcare Bio-Sciences Corp., Piscataway NJ, USA). After washing 5×, the plates were developed with 100 µl/well *o*-phenylenediamine in phosphate-citrate buffer (Sigma-Aldrich), stopped with 100 µl/well 1 N H_2_SO_4_ (VWR, West Chester PA, USA) and read at 490/620 nm.

### Mimotope analysis

Phage insert sequences were analyzed using the computer program 3DEX [52]. After checking for linear homology to SHIV-1157ip gp160 [33], the peptide sequences were compared to published PDB structure files of HIV-1 gp120 [32], [53] to identify conformational homology. Phages were grouped according to their peptide motifs, and selected phages were used for further analysis and immunization studies.

### Immunization of mice

Selected recombinant phages were grouped according to motifs; each group consisted of 5–6 phages with similar but not identical peptide sequences. Phages of each group were combined and used as five different mixtures for immunization. Mice were primed once with SHIV-1157ip *env* DNA (intramuscularly; 100 µg in 100 µl PBS) and boosted subcutaneously (s.c.) with 10^12^ phage particles in 100 µl PBS/MPL (Sigma-Aldrich) every 4–5 weeks. Serum samples were collected 2–5 weeks after each boost. After four phage boosts, serum samples were tested for their neutralizing capacity against HIV-1_SF162.LS_ and SHIV-1157ip. In a pilot study, 11 mice were given an additional boost with trimeric SHIV-1157ip gp160 (s.c.; 20 µg in 100 µl PBS with incomplete Freund's adjuvant (IFA) (Sigma-Aldrich) and their sera were tested for the presence of neutralizing antibodies.

### Neutralization assay

SHIV-1175ip was prepared in rhesus monkey PBMC, HIV pIndieC was prepared in human PBMC and HIV_SF162.LS_ pseudovirus (kindly provided by David Montefiori) was prepared using cotransfection of 293T cells with an *env* expression plasmid and Δenv backbone vector. TZM-bl cells encode the luciferase gene under the control of the HIV-1 promoter; both CD4 and CCR5 are also expressed on the cell surface (AIDS Research and Reference Program, Division of AIDS, NIAID, NIH). A total of 5,000 cells/well were seeded overnight in 100 µl DMEM/10% FCS (Gibco-Invitrogen, Grand Island NY, USA). Serial 2-fold dilutions of immune sera were prepared in triplicates in 96-well round-bottom plates (Becton Dickenson, Franklin Lakes NJ, USA). In parallel, the pre-immune sera were serially diluted and used as controls. “Virus only” wells received 50 µl medium. The virus was diluted (1∶500 for SHIV-1157ip: 36 ng/ml p27; 1∶500 for HIV pIndieC: 35 ng/ml p24; 1∶300 for HIV_SF162.LS_: 300 ng/ml p24) and 50 µl of virus was added to all wells. The plate was incubated for 1 h at 37°C in 5% CO_2_, after which time 10 µl of a 400 µg/ml DEAE-Dextran solution (Sigma-Aldrich) was added to all wells and the entire mixture was transferred into the 96-well flat–bottom plate with the seeded TZM-bl cells. The next day, medium was replaced with fresh medium and incubated another 24 h. Bright-Glo luciferase substrate (Promega, Madison WI, USA) was added to the plate the following day and luciferase activity was measured. The percent neutralization was calculated using the following equation:
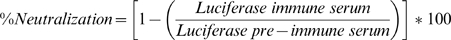



### Dot spot analysis

Trimeric SHIV-1157ip gp160 was spotted onto a nitrocellulose membrane (Whatman GmbH, Dassel, Germany) (50 ng/spot in native or reduced conditions (10 mM TCEP (Pierce, Rockford IL, USA); 1% SDS (Sigma-Aldrich); boiled for 2 min). Strips were blocked with PBSCT for 1 h at room temperature and incubated overnight at 4°C with antibodies (diluted in PBSCT). The next day, strips were washed 3× with PBST and incubated with anti-monkey IgG HRP-conjugate (1∶2,000 in PBSCT; Sigma-Aldrich). After 1 h at room temperature, strips were washed 5× with PBST and developed using Opti-4CN substrate (Bio-Rad Laboratories, Hercules CA, USA).

### ELISA

Plates were coated overnight at 4°C with 100 ng/well native or reduced (10 mM TCEP/1% SDS; boiled for 2 min) proteins in 100 µl coating buffer. After washing 3×, plates were blocked with 200 µl/well PBSCT for 1 h at room temperature and then incubated overnight at 4°C with mouse serum (1∶175) or rhesus monkey serum (1∶800; 100 µl/well diluted in PBSCT). Plates were washed 3× and incubated with HRP-conjugated antibodies (1∶2,000 in PBSCT, 100 µl/well) for 1 h at room temperature. After washing 5×, plates were developed using 100 µl/well *o*-phenylenediamine in phosphate-citrate buffer, stopped with 100 µl/well 1 N H_2_SO_4_ and read at 490/620 nm.

### Mimotope fusion proteins

Fusion proteins were cloned into the expression vector pPEPTIDE according to manufacturer's instructions (MoBiTec GmbH, Göttingen, Germany). Briefly, phage peptide insert were amplified using two primers (pPeptide-rev: 5′-GGCCCGGGGATCCTAACTTTCAACAGTTTCGGCCGAACCTCCACC; pPeptide-fw: 5′-CGCCCGCGGATTAATGGCCCTTTAGTGGTACCTTTCTATTCTCACTCT) to introduce the underlined cloning restriction sites, digested with AseI and BamHI (Fermentas Inc., Glen Burnie MD, USA) and gel-purified using NucleoSpin Extract II (Macherey-Nagel Inc., Bethlehem PA, USA). Six ng of mimotope DNA were ligated with 100 ng vector and transformed into BL21(DE3) cells (Novagen, Madison WI, USA). After sequence analysis, proteins were expressed according to the manual and purified using standard chromatography using the Ni-charged Profinity IMAC resin (Bio-Rad). Of note, the vector encodes a His tag to assess fusion protein expression. The plasmid is based on the pET expression vector system and induction of the T7 promoter leads to the expression of a highly expressed fusion protein (89 aa) followed by a higher affinity poly(His) region (53 aa) for purification and the C-terminal mimotope. The expressed mimotope fusion protein migrates at a size of around 26 kD.

### Western blot

Standard SDS PAGE was performed with 40 µg protein per gel (Bio-Rad). Protein was transferred onto a nitrocellulose membrane (Bio-Rad) using a wet blot apparatus (Bio-Rad). The membrane was cut into strips, blocked with 3% PBSCT and individual strips were incubated with the appropriate serum (1∶200) or antibodies (1∶1,000 in 3% PBSCT) overnight at 4°C. Membranes were washed 3× and incubated for 1 h at room temperature with an HRP-conjugated antibody (1∶2,000 in 3% PBSCT). After washing 5×, the strips were developed with Opti-4CN substrate (Bio-Rad).

### Statistical analysis

Statistics were calculated using a two-tailed paired t test and only applied to matching pairs of mice comparing the significance between the post-phage boosts and post-gp160 boost (GraphPad Prism 5 for Windows, GraphPad Software).

## Supporting Information

Figure S1 Cross-reactivity profile of mimotope fusion proteins by ELISA. Mimotope fusion proteins were tested by ELISA for cross-recognition with 9 SHIV-positive monkey sera from our cohort. Two naïve sera (CF12, RJi-2) and a fusion protein without mimotope insert (pPeptide) were used as negative controls.
(0.94 MB DOC)

Figure S2 Serum reactivity with AIV12.4. The linear mimotope fusion protein AIV12.4 was tested for reactivity with various RKl-8 sera by ELISA. As negative controls, two naïve RM sera (CF12, RJi-2) were included.
(1.30 MB DOC)

Figure S3 Analysis of immune mouse sera after DNA prime/phage boosting or DNA prime/phage+gp160 boosting. (A) Anti-Env titers. Reciprocal serum dilution of each mouse for the post-phage boosts and post-gp160 boost is shown. Red symbols indicate 3rd vs. 5th bleed, green symbol indicates no matching 5th bleed, both due to serum restrictions. (B) Neutralization with mouse immune sera. Bleeds were tested for 50% neutralization (IC50) against homologous SHIV-1157ip and heterologous HIV-1SF162.LS. Post-phage boosts (triangles) are compared to post-gp160 boost (squares). (C, D) Vaccination-induced antibody responses against conformational and linear mimotopes. Mimotopes were cloned and expressed as fusion proteins. The latter were used to test whether DNA priming/phage boosting (triangles) or DNA priming/phage+gp160 boosting (squares) of mice had induced antibodies against the original phage-encoded peptide mimotopes. Sera from two selected mice were tested for reactivity to each of the mimotopes used in the immunization mixture. (C) Mouse #1.4, immunized with potential conformational V3-loop mimotopes. (D) Mouse #2.5, immunized with linear C-terminal mimotopes.
(0.91 MB DOC)
