# Supplementary figures and images for: Inducing Cross-Clade Neutralizing Antibodies against HIV-1 by Immunofocusing
Source: PLoS One. 2008 Dec 15;3(12):e3937. doi: 10.1371/journal.pone.0003937 (PMC2597739; doi:10.1371/journal.pone.0003937)

**Figure S1**

**
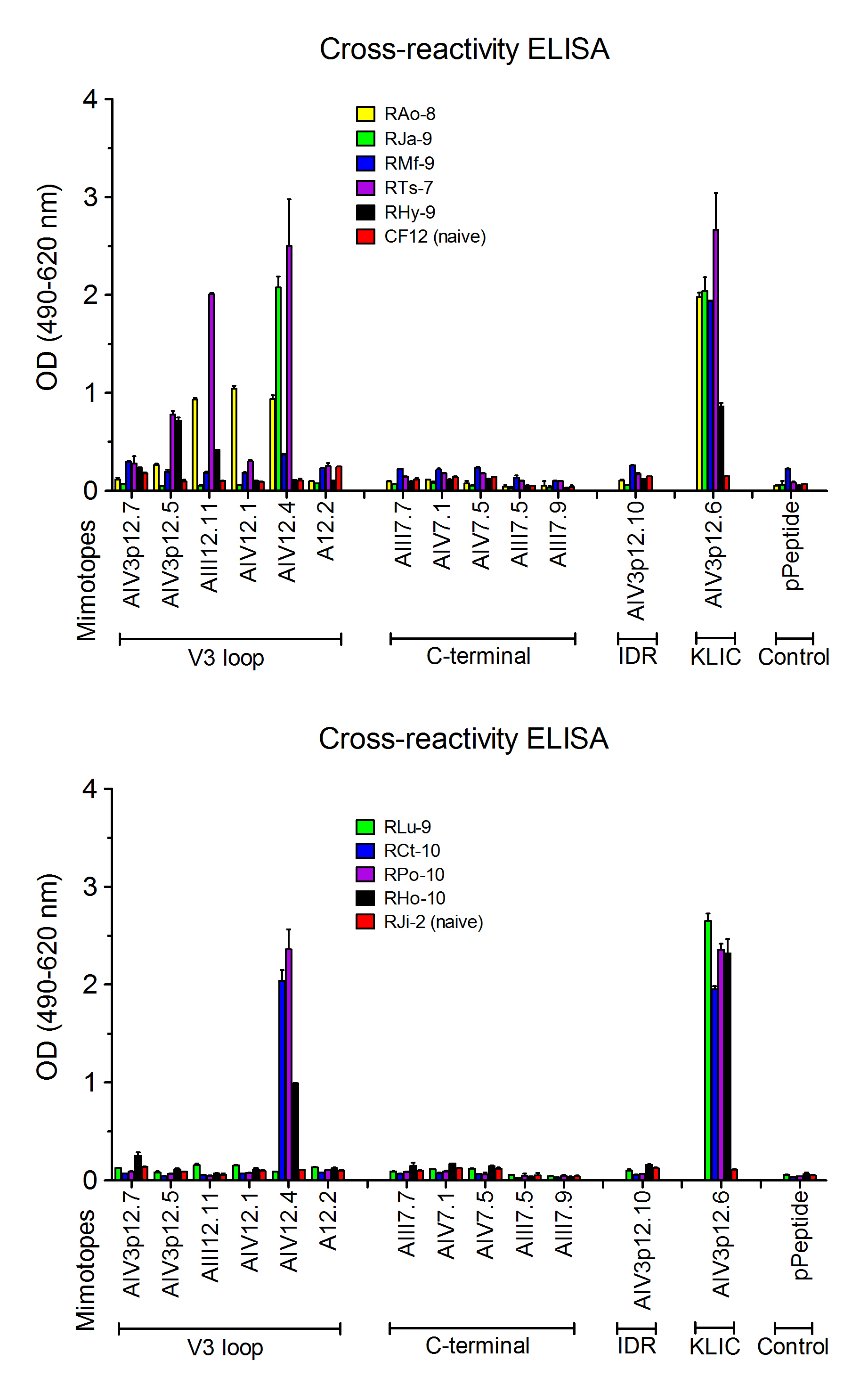
**

Supplement: Figure S1 — Cross-reactivity profile of mimotope fusion proteins by ELISA. Mimotope fusion proteins were tested by ELISA for cross-recognition with 9 SHIV-positive monkey sera from our cohort. Two naïve sera (CF12, RJi-2) and a fusion protein without mimotope insert (pPeptide) were used as negative controls. (0.94 MB DOC) [file pone.0003937.s001.doc]

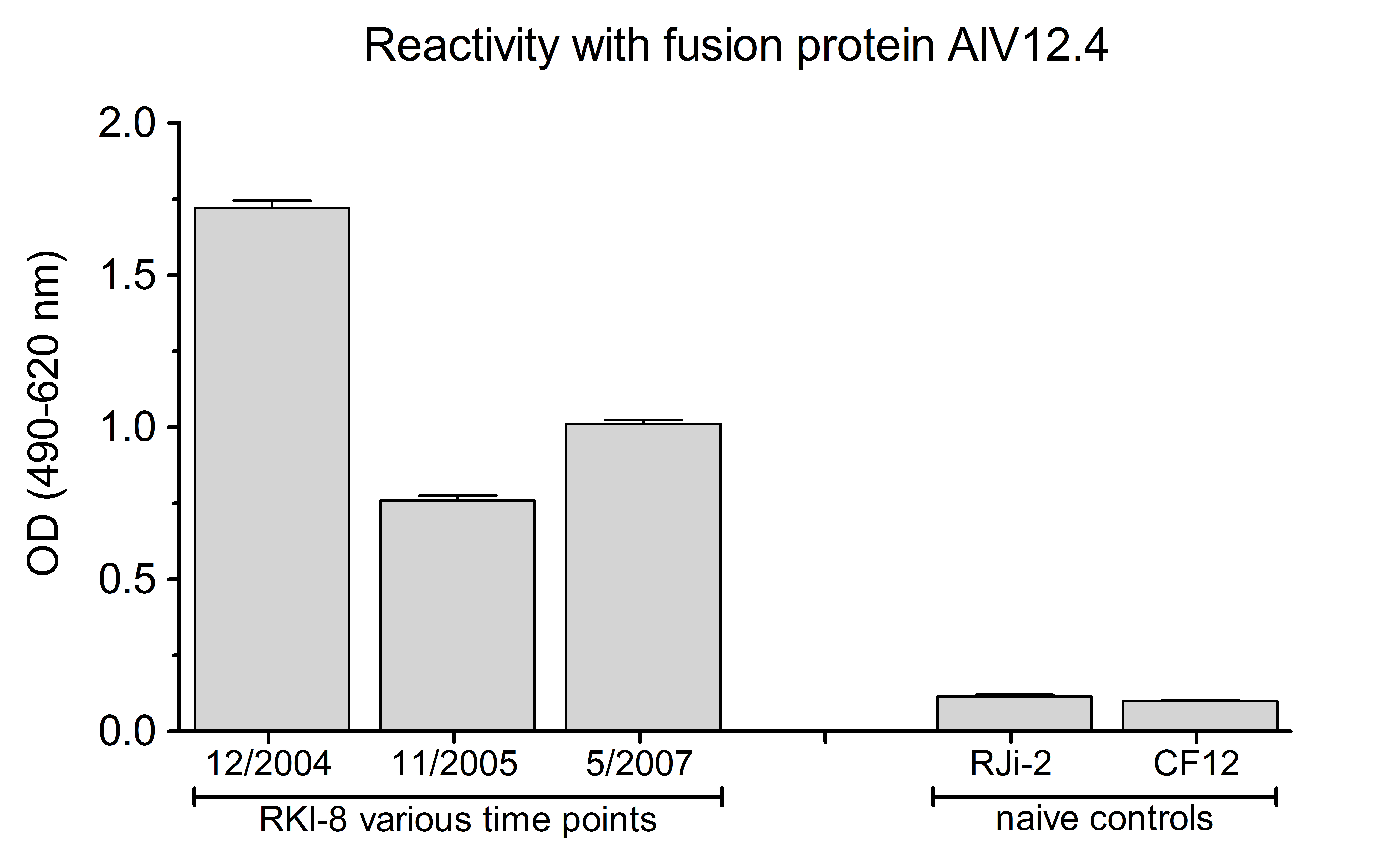
**Figure S2**

Supplement: Figure S2 — Serum reactivity with AIV12.4. The linear mimotope fusion protein AIV12.4 was tested for reactivity with various RKl-8 sera by ELISA. As negative controls, two naïve RM sera (CF12, RJi-2) were included. (1.30 MB DOC) [file pone.0003937.s002.doc]

**Figure S3**

**
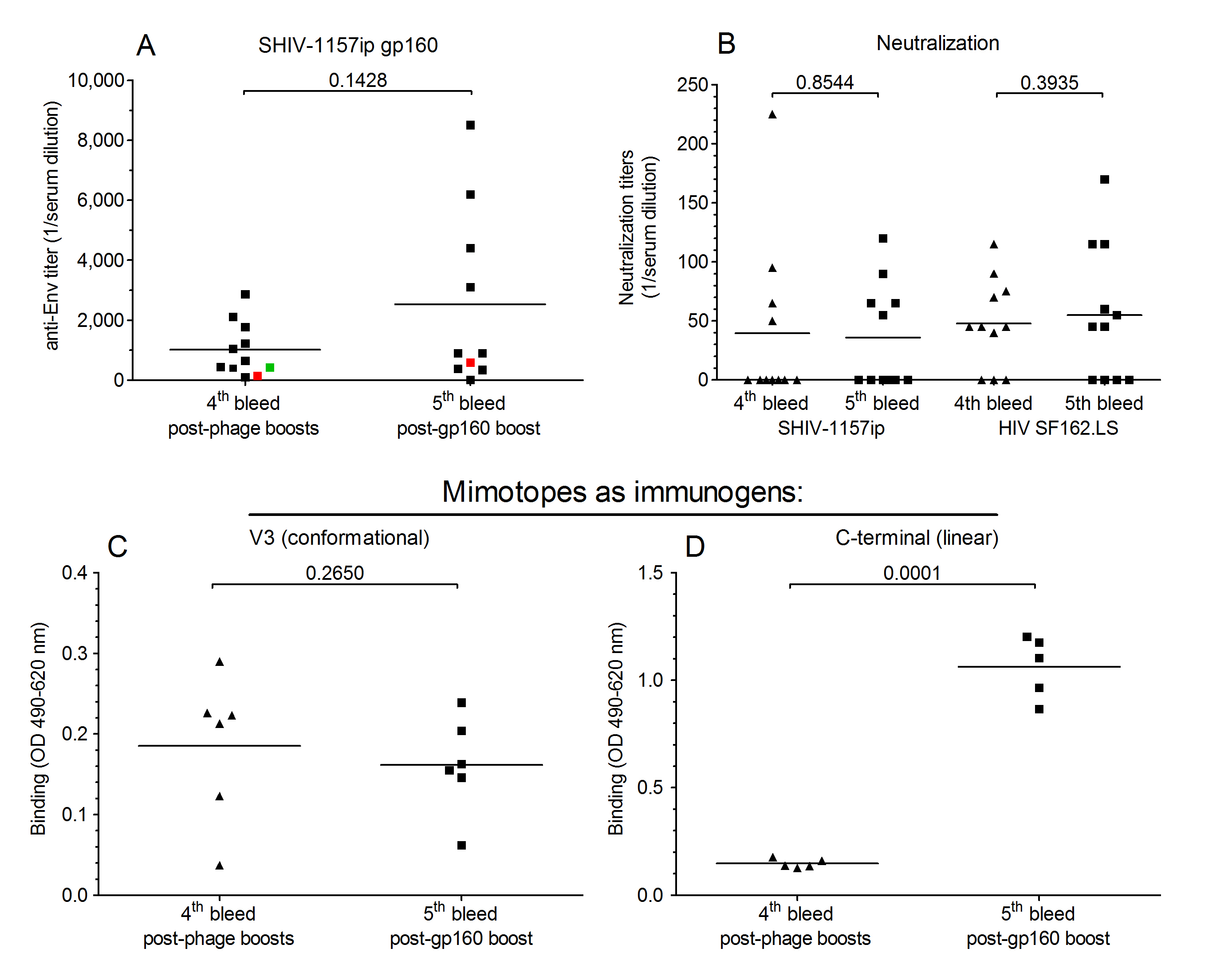
**

Supplement: Figure S3 — Analysis of immune mouse sera after DNA prime/phage boosting or DNA prime/phage+gp160 boosting. (A) Anti-Env titers. Reciprocal serum dilution of each mouse for the post-phage boosts and post-gp160 boost is shown. Red symbols indicate 3rd vs. 5th bleed, green symbol indicates no matching 5th bleed, both due to serum restrictions. (B) Neutralization with mouse immune sera. Bleeds were tested for 50% neutralization (IC50) against homologous SHIV-1157ip and heterologous HIV-1SF162.LS. Post-phage boosts (triangles) are compared to post-gp160 boost (squares). (C, D) Vaccination-induced antibody responses against conformational and linear mimotopes. Mimotopes were cloned and expressed as fusion proteins. The latter were used to test whether DNA priming/phage boosting (triangles) or DNA priming/phage+gp160 boosting (squares) of mice had induced antibodies against the original phage-encoded peptide mimotopes. Sera from two selected mice were tested for reactivity to each of the mimotopes used in the immunization mixture. (C) Mouse #1.4, immunized with potential conformational V3-loop mimotopes. (D) Mouse #2.5, immunized with linear C-terminal mimotopes. (0.91 MB DOC) [file pone.0003937.s003.doc]
